# Polysaccharide Isolated From *Tetrastigma hemsleyanum* Activates TLR4 in Macrophage Cell Lines and Enhances Immune Responses in OVA-Immunized and LLC-Bearing Mouse Models

**DOI:** 10.3389/fphar.2021.609059

**Published:** 2021-03-24

**Authors:** Fang-mei Zhou, Yu-chi Chen, Chao-ying Jin, Chao-dong Qian, Bing-qi Zhu, Ying Zhou, Zhi-shan Ding, Yi-qi Wang

**Affiliations:** ^1^ College of Medical Technology, Zhejiang Chinese Medical University, Hangzhou, China; ^2^ School of Pharmaceutical Science, Zhejiang Chinese Medical University, Hangzhou, China; ^3^ College of Life Science, Zhejiang Chinese Medical University, Hangzhou, China; ^4^ Department of Cardiology, Zhejiang Provincial People's Hospital, People's Hospital of Hangzhou Medical College, Hangzhou, China

**Keywords:** polysaccharide, TLR4, RAW264.7, THP-1, adjuvant, antitumor, *Tetrastigma hemsleyanum*

## Abstract

*Tetrastigma* *hemsleyanum* Diels et Gilg is a valuable Chinese medicinal herb with a long history of clinical application. Our previous study isolated and characterized a purified polysaccharide from the aerial part of *Tetrastigma hemsleyanum* (SYQP) and found it having antipyretic and antitumor effects in mice. A preliminary mechanistic study suggests these effects may be related to the binding of toll-like receptor (TLR4). The objective of this study is to further explore the detailed stimulating characteristics of SYQP on TLR4 signaling pathway and its *in vivo* immune regulating effect. We use HEK-BLUE hTLR4, mouse and human macrophage cell lines, as research tools. *In vitro* results show SYQP activated HEK-BLUE hTLR4 instead of HEK-BLUE Null cells. The secretion and the mRNA expression of cytokines related to TLR4 signaling significantly increased after SYQP treatment in both PMA-induced THP-1 and RAW264.7 macrophage cell lines. The TLR4 antagonist TAK-242 can almost completely abolish this activation. Furthermore, molecules such as IRAK1, NF-κB, MAPKs, and IRF3 in both the MyD88 and TRIF branches were all activated without pathway selection. *In vivo* results show SYQP enhanced antigen-specific spleen lymphocyte proliferation and serum IgG levels in OVA-immunized C57BL/6 mice. Orally administered 200 mg/kg SYQP induced obvious tumor regression, spleen weight increase, and the upregulation of the mRNA expression of TLR4-related cytokines in Lewis lung carcinoma–bearing mice. These results indicate SYQP can act as both a human and mouse TLR4 agonist and enhance immune responses in mice (*p* < 0.05). This study provides a basis for the development and utilization of SYQP as a new type of TLR4 agonist in the future.

## Introduction

The toll-like receptor (TLR) family is a class of pattern-recognition receptors of mammalian species, which can specifically identify conservative molecules of pathogenic microorganisms and regulate the natural and acquired immune responses of organisms (Akira and Takeda, 2004). TLR4 was the first member to be found, and it is unique for its ability to induce both MyD88-dependent and -independent (TRIF) pathways. Activation of TLR4 receptor on antigen presenting cells can promote the uptake, processing, and presentation of foreign antigens, which is helpful to activate T cells and enhance the antigen-specific immune response. Therefore, TLR4 agonists can be developed as immune adjuvants to enhance the immune responses to vaccines (Reed et al., 2016). Recent studies show that TLR4 agonists can also break tumor-induced immune tolerance and enhance the innate and adaptive immune responses against cancer (Boushehri Shetab and Lamprecht, 2018). The activation of the MyD88-dependent pathway results in the secretion of inflammatory cytokines such as TNF-α, which has been shown to serve as a neoadjuvant for local chemotherapy and an enhancer of the intratumoral penetration of anticancer drugs (Boushehri Shetab and Lamprecht, 2018). Activation of the TRIF pathway can induce the phosphorylation of IRF3 and its translocation to the nucleus, which is required for the expression of type I IFNs and IP-10 (Interferon-inducible protein-10). IP-10 is a key chemokine recruiting effector T cells to the tumor microenvironment. Type I IFNs can not only further promote the secretion of IP-10 but also upregulate the expression of MHCⅠ on tumor cells, thus enhancing the antitumor CD8^+^ T cell effector response (Najbauer et al., 2019). Therefore, TLR4 receptor agonists with the ability to activate both MyD88-dependent and -independent pathways are also promising drugs for cancer immunotherapy.

LPS (endotoxin), which is the main component of the outer membrane of Gram-negative bacteria, is the primary natural ligand of TLR4 (Park et al., 2009). Animal experiments and clinical trials suggest that the subcutaneous injection of LPS has strong immune adjuvant activity and the intravenous injection has an immunotherapeutic effect on some tumors (Otto et al., 1996; Toussi and Massari, 2014; Han et al., 2017; Melssen et al., 2019). However, symptoms such as fever, myalgia, and chills occurred in most patients because of the excessive release of inflammatory cytokines. Although chemical modification has been performed to develop safer LPS derivatives/synthetic analogues, most of them are still hampered by the remarkable systemic side effects. Up to now, only MPLA (monophosphoryl lipid A), a detoxified form of LPS, has been approved to be applied as an immune adjuvant in the clinic (Casella and Mitchell, 2008). Therefore, finding new types of safe and effective TLR4 agonists is still an urgent task at present.

Polysaccharides from natural plants have recently attracted lots of attention due to their definite biological activities, especially their immune-regulating and antitumor effects. Many polysaccharides from natural traditional Chinese medicinal plants are found to regulate the immune function by activating the TLR4 signaling pathway and show antitumor activities (Zhou et al., 2017; Xie et al., 2019). As these medicinal plants have been confirmed to be safe based on years of clinical application, they have become a good source for finding new TLR4 agonists. *Tetrastigma hemsleyanum* Diels et Gilg belongs to the grape family Vitaceae and is a valuable Chinese medicinal herb mainly distributed in the south of China. It was traditionally used to treat cancer and infection, especially respiratory diseases in the clinic. According to folk medicine of China, it is even prepared as a tea drink for health care and immunity enhancement. Long-term application practice in humans shows that the plant is safe and almost nontoxic. Although the activities of some active components in the plant, such as flavonoids, phenols, and isoquercitrin, have been reported (Xia et al., 2018; Ji et al., 2019; Wang et al., 2019), the characteristic immune-regulating activity of polysaccharides from *Tetrastigma hemsleyanum* is still unclear. Our previous study isolated and characterized a purified polysaccharide from the aerial part of *Tetrastigma hemsleyanum* (SYQP) and found it has antipyretic and antitumor effects in mice (Zhu et al., 2020). A preliminary mechanistic study suggests these effects may be related to the binding of TLR4. In this study, we used the mouse macrophage cell line RAW264.7 and the phorbol 12-myristate 13-acetate (PMA)–stimulated human monocyte cell line THP-1 (can be differentiated into macrophages) to further explore the detailed characteristics of the effects of SYQP on TLR4 signaling pathways in both human and mouse cell lines. In addition, we used OVA-immunized mice and a Lewis lung cancer (LLC) mouse model to determine whether SYQP can enhance immune responses and show antitumor activity *in vivo*.

## Materials and Methods

### Materials and Chemicals

The aerial parts of *Tetrastigma hemsleyanum* Diels et Gilg were obtained from Hangzhou China Agrotime Agri-Tech Co., Ltd. The plant was authenticated by one of the authors (Prof. Zhi-Shan Ding), and a voucher specimen was deposited in the College of Medical Technology, Zhejiang Chinese Medical University, China. DEAE-52 and Sephadex G-200 were purchased from Shanghai YuanYe Bio-Technology Co. Ltd. (Shanghai, China). A Pierce LAL Chromogenic Endotoxin Quantitation Kit was purchased from Thermo Fisher Scientific (CA, United States). A CellTiter 96^®^ AQueous One Solution Cell Proliferation Assay (MTS) was purchased from Promega Corporation (WI, United States), and lipopolysaccharide (LPS) and PMA were purchased from Sigma Chemical Co. (MO, United States); TAK-242 was purchased from MedChem Express (MCE) (NJ, United States); the mouse TNF-α ELISA kit was purchased from Thermo Fisher Scientific (CA, United States). Antibodies against IRAK1, phospho-IRF3 (Ser396), IRF3, phospho-IKKα/β (Ser176/180), IKKβ, phospho-NF-κB p65 (Ser536), NF-κB p65, phospho-JNK (Thr183/Tyr185), JNK, phospho-ERK (Thr202/Tyr204), ERK, phospho-p38 (Thr180/Tyr182), p38, and β-actin were purchased from Cell Signaling Technology (MA, United States). Goat Anti-Mouse IgG peroxidase conjugate and Goat Anti-Rabbit IgG peroxidase conjugate were purchased from Jackson ImmunoResearch (PA, United States). PrimeScript RT reagent Kit, RNAiso Plus, and SYBR Premix Ex Taq II were purchased from Takara Biotechnology (Shiga, Japan). ACK Lysis Buffer was purchased from Beyotime Biotechnology (Shanghai, China).

### Extraction and Purification of SYQP

SYQP was prepared and characterized in our laboratory as previously reported (Zhu et al., 2020). Briefly, the dried aboveground parts of *Tetrastigma hemsleyanum* were ground into fine powders and extracted with distilled water under reflux for 4 h. The water extract was filtered and centrifuged, followed by concentration under vacuum. The concentrated water extract was added to 95% ethanol solution and subsequently placed at 4°C for 12 h to precipitate polysaccharides. The crude polysaccharides were deproteinized, concentrated, and loaded on a DEAE-Sepharose fast flow column and Superdex-200 chromatography column to obtain SYQP. Molecular weight was determined using high performance gel permeation chromatography (HPGPC) on a Water Ultrahydrogel 500 Column (Milford, MA, United States). Neutral sugar content and uronic acid content were respectively determined by phenol-sulfuric acid colorimetry method, and monosaccharide composition was analyzed by Blumenkrantz and Asboe-Hansen method. Monosaccharide composition of SYQP was assessed by a gas chromatography mass spectrometry, and infrared spectral analysis was conducted on a Nicolet iS5 Fourier transform infrared (FTIR) spectrometer (Waltham, MA, United States).

### Cell Culture

HEK-BLUE hTLR4 and HEK-BLUE Null cells were kindly provided by Professor Thomas C. Mitchell (University of Louisville, United States). The mouse macrophage cell line (RAW264.7) and human monocytic leukemia cell line (THP-1) were purchased from ATCC (American type culture collection). 3^##^ to 30^##^ passages of cells were used in this study. All of the cells were cultured in Dulbecco’s Modified Eagle Medium (DMEM) containing 10% heat-inactivated fetal bovine serum, supplemented with 100 units/ml penicillin and 100 mg/ml streptomycin at 37°C and 5% CO_2_. Thp-1 monocytes were differentiated into macrophages by phorbol 12-myristate 13-acetate (PMA) as described previously with a few modification (Park et al., 2007). The suspension THP-1 cells in logarithmic growth phase were collected, and the cell number was adjusted to 500,000/ml. 5 ng/ml PMA was added into the cell suspension. Blow and mix with pipettes and plate the cells in 96 microplate with 100 μl cell suspension per well (50,000 cells/well). After 48 h, the cells adhered well and grew out with protuberance. Drug treatment can be done at this time.

### Cytotoxicity Evaluation by MTS Assay

HEK-BLUE hTLR4, HEK-BLUE Null, RAW264.7, or THP-1 cells were seeded in a 96-well plate containing 5 × 10^4^ cells per well. HEK-BLUE hTLR4, HEK-BLUE Null, and RAW264.7 cells were cultured for 2 h to adapt to the environment. THP-1 cells were treated by 5 ng/ml PMA for 48 h to promote THP-1 monocyte differentiation into macrophages. After that, cell culture medium containing SYQP at various concentrations (0.1–1,000 μg/ml) was added to each well. After 24 h of incubation, 20 μl of MTS solution was added to each well with 100 μl of medium, and the plate was further incubated at 37°C for 2 h. Finally, the absorbance at 490 nm of the formazan was assessed using a microplate reader. Cell viability (%) = [(OD _treatment_ − OD _blank_)/(OD _control_ − OD _blank_)] × 100%

### Detection of NF-κB Activity in HEK-BLUE hTLR4 and HEK-BLUE Null Cells

The cells in the logarithmic growth phase were collected and plated into 96-well plates with 5 × 10^4^ cells per well. SYQP 0.0001 – 100 μg/ml was added to the HEK-BLUE hTLR4 or HEK-BLUE Null cells to observe the effect of SYQP on NF-κB activation of the cells. Combined administration of 1 μg/ml LPS and 0.0001 – 100 μg/ml SYQP was performed in the HEK-BLUE hTLR4 cells to observe the inhibition of SYQP on LPS-induced NF-κB activation. After 24 h of treatment, 60 μl of cell culture supernatant was mixed with 140 μl of QUANTI-Blue™ solution containing alkaline phosphatase substrate. The mixtures were incubated at 37°C for 30 min, and the OD value was read at 620 nm. The NF-κB activation of the cells was calculated by measuring the content of secretory alkaline phosphatase in the cell culture supernatant. The activation degree of NF-κB, which to some extent represents the degree of activation of TLR4, was calculated by comparing it with the 1 μg/ml LPS-treated group (this concentration of LPS results in maximum activation of TLR4). Activation degree of NF-κB = (OD _treatment_ − OD _blank_)/(OD _1 μg/ml LPS_ − OD _blank_).

### TNF-α Measurement by ELISA

RAW264.7 cells or THP-1 cells were seeded in a 96-well plate containing 5 × 10^4^ cells per well. SYQP at 0.1–500 μg/ml was added to the cell supernatants. Cells treated with medium without drugs were set as the negative control, and cells treated with 1 μg/ml LPS were set as the positive control. After 24 h of treatment, culture supernatants were collected for the detection of TNF-α levels using commercial ELISA kits in accordance with the manufacturer’s instructions.

### Real-Time PCR Analysis

RAW264.7 cells or THP-1 cells were seeded in a 6-well plate containing 1 × 10^6^ cells per well. Cell culture medium containing SYQP at various concentrations (1, 10, 100, or 500 μg/ml) was added to each well. Cells treated with medium without drugs were set as the negative control group, and cells treated with 1 μg/ml LPS were set as the positive control group. After 6 h of treatment, total RNA was extracted with RNAiso Plus, and SuperScript II was used for the reverse transcription of total RNA. The total RNA of the spleen tissue of LLC bearing mice was also extracted and do the reverse transcription. A CFX96 Touch™ Real-Time PCR Detection System (Bio-Rad) was used to amplify cDNA, and SYBR Green PCR Master Mix was used to determine the quantity of mRNA. The sequences of the primers used in the PCRs are presented in Supplementary Table S1. All of the experimental procedures were in accordance with the manufacturer’s protocols. Relative quantification of samples according to the housekeeping gene GAPDH was achieved by the 2^−△△Ct^ method.

### Western Blot Analysis

RAW264.7 cells were seeded in a six-well plate containing 1 × 10^6^ cells per well. After treatment with various concentrations of SYQP (1–500 μg/ml) for 2 h (TLR4, IRAK1, IRF3), 30 min (IKK, NF-κB, p38, JNK, and ERK) or treatment with 100 μg/ml SYQP for 30, 60, 90, and 120 min, cells were washed twice with cold PBS and lysed by a lysis buffer. In order to observe the effect of TLR4 inhibitor on the activation of TLR4 signaling, the RAW264.7 cells were pretreated with TAK-242 20 μg/ml for 30 min, then SYQP 100 μg/ml or LPS 1 μg/ml were added into the cell supernatant alone or combined with TAK-242 for another 2 h. After that the cells were lysed and the protein of cell lysates was quantified by the BCA reagent. Harvested proteins were denatured at 100°C for 10 min, and 50 μg of protein from each sample was electrophoresed by sodium dodecyl sulfate polyacrylamide gel electrophoresis (SDS-PAGE) and then transferred onto polyvinylidene difluoride membranes. After that, the membranes were blocked for 1 h at room temperature in 5% nonfat dry milk, followed by 3 h of incubation with primary antibody at room temperature. Horseradish peroxidase (HRP)–conjugated secondary antibody was incubated for 1 h at room temperature. Finally, the signal was visualized with the ECL Detection Kit.

### Immunization

Six-week-old female C57BL/6 mice were purchased from the Medical Animal Center, Zhejiang Chinese Medical University, Zhejiang Province, China. The ethical approval number for the animal model study is SYXK 2018-0012. All animals were housed under standard laboratory conditions. The temperature was controlled at 24 ± 1°C and humidity at 50 ± 10%. All experimental procedures conformed to the People’s Republic of China (PRC) guidelines for the Care and Use of Laboratory Animals and were carried out strictly in accordance with the Guidelines of Zhejiang Chinese Medical University for Animal Experiments. Animal immunization was performed as described previously (Sun et al., 2009). All of the mice were immunized twice by being subcutaneously injected (s.c.) with OVA (100 µg) at weeks 0 and 3. Saline (s.c.) administration was used as a nonimmunized normal control. SYQP (100 or 200 mg/kg) was given once a day for four consecutive days before each immunization. Blood sera and splenocytes were collected 2 weeks after the second immunization for measurement of OVA-specific antibody and lymphocyte proliferation.

### Determination of Serum IgG

Serum OVA-specific IgG was measured by ELISA as described previously (Xiao et al., 2007). Briefly, 96-well microtiter plates were coated in 100 μl of OVA solution (5 μg/ml in 0.05 M carbonate buffer, pH 9.6) and incubated overnight at 4°C. Then, the plates were washed three times with PBS containing 0.05% Tween 20 (PBS/Tween) and blocked with 5% FBS (200 μl/well) at 37°C for 2 h. To measure the level of IgG, diluted serum (1:800) was added, and the plates were incubated at 37°C for 1 h. After another washing, goat anti-mouse IgG was added, and the plates were incubated again at 37°C for 1 h. The plates were washed again, and TMB was added to each well and incubated for 15 min in the dark. The reaction was stopped using 50 μl of 2 M H_2_SO_4_. The optical density of the plates was read at 450 nm by microplate reader.

### Splenocyte Proliferation Assay

Splenocyte proliferation was assayed as described previously with a few modification (Su et al., 2012). The spleen was collected 2 weeks after the second immunization. The organ was minced and passed through a steel mesh to obtain a homogeneous cell suspension. To lyse erythrocytes, ACK buffer was added. After centrifugation (1,400 rap/min for 5 min), the pelleted cells were washed in PBS and resuspended in RPMI 1640 supplemented with 100 IU/ml penicillin, 100 μg/ml streptomycin, and 10% heat-inactivated FBS. Cell numbers were counted with a hemocytometer by the trypan blue dye exclusion technique. Cells were seeded into 96-well plates at 5 × 10^5^ cells/well. OVA at 100 μg/ml was added to stimulate the proliferation of the splenocytes for 48 h. MTS solution was then added to each well. After incubation for 2 h, the absorbance at 490 nm was assessed using a microplate reader. Stimulation Index (SI) was calculated according to the following formula: SI = (OD _stimulated well_ − OD _blank well_)/(OD _unstimulated well_ − OD _blank well_).

### Establishment of Lewis Lung Cancer–Bearing Mouse Model

C57BL/6 mice were implanted s.c. with the Lewis lung carcinoma (LLC) cell line (1 × 10^6^/mouse). On the seventh day after implantation, mice with palpable tumors were orally administered daily by gavage with SYQP (100 or 200 mg/kg) for seven consecutive days. An equal volume of saline was orally administered as the control. DDP (2 mg/kg) was intraperitoneally injected as the positive control once every other day. At the end of the administration, mice were sacrificed, and tumor tissue and spleen were separated and weighed.

### Elimination of Endotoxin Contamination

All of the SYQP samples were examined for the presence of endotoxin by an LAL kit according to the instructions of the manufacturer. Polymyxin B (PMB) is a potent antibiotic that binds to and neutralizes LPS (Cardoso et al., 2007). In this study, 5 μg/ml PMB was combined treatment with LPS 1 μg/ml or SYQP 100 μg/ml on HEK-BLUE hTLR4 cells.

### Statistical Analysis

The data were expressed as the mean ± standard deviation (SD). All data were analyzed with GraphPad Prism 8.0 software. Comparisons between groups were performed using one-way ANOVA, followed by Tukey’s multiple comparisons test; ^*^ *p* < 0.05, **p < 0.01, and ***p < 0.001 were considered to be statistically significant.

## Results

### Chemical Characterization of SYQP

In the purification of the polysaccharides obtained from *Tetrastigma hemsleyanum*, gradient elution was carried out and a relatively strong peak was observed in the fraction eluted by 0.2 mol/L NaCl solution as shown in Supplemental Figure S1. A further purification by Superdex-200 exhibited a single peak, which was collected as SYQP for further study. Chemical characterization was conducted to this SYQP, whose molecular weight was determined to be 66.2 kDa by HPGPC and total sugar uronic acid contents were determined to be 83.3 and 48.9%. SYQP was mainly composed of galacturonic acid (GalA), glucose (Glc), mannose (Man), arabinose (Ara), galactose (Gal), and rhamnose (Rha) in the molar ratio of 11.3:7.1:2.5:1.0:0.9:0.5, indicating a relatively high content of GalA, after comparing the composition of SYQP before and after reduction as shown in Supplemental Figure S1. FTIR results demonstrated the presence of carboxyl groups according to the absorption at 1,618.34 cm^−1^ and the peak at 1,745.16 cm^−1^ corresponded to the existence of uronic acid. Other peaks at 3,398.46 cm^−1^ and 2,945.73 cm^−1^ were related to O-H stretch of carbohydrates and C-H stretching.

### SYQP Stimulated Human TLR4 in HEK-BLUE hTLR4 Cells

**FIGURE 1:**
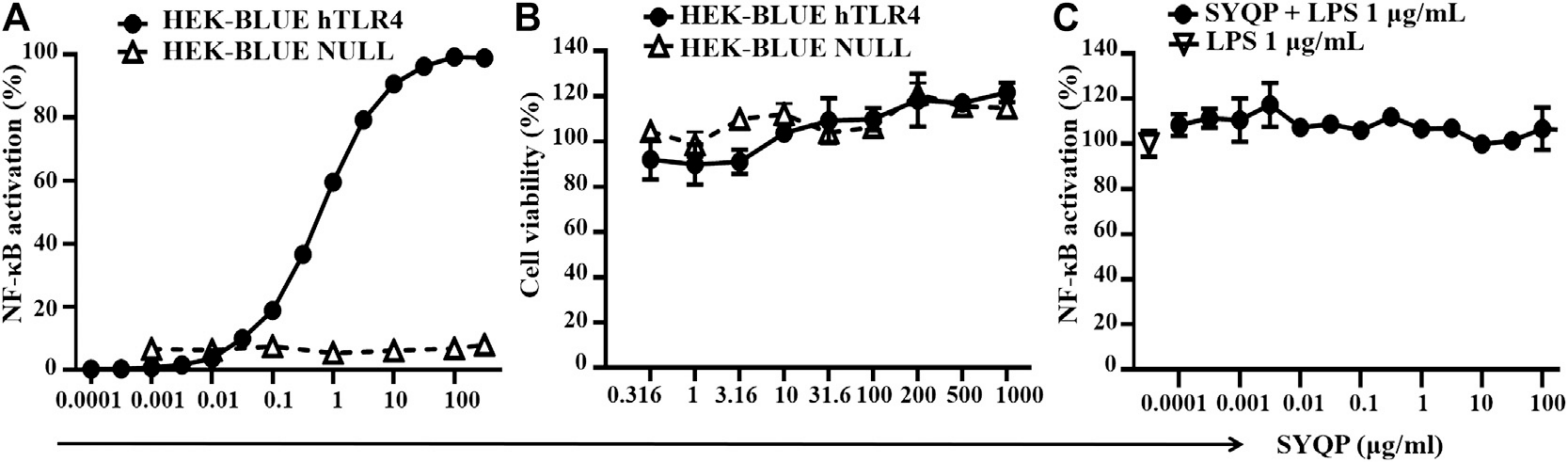
SYQP stimulated human TLR4 in HEK-BLUE hTLR4 cells. **(A)** SYQP could activate NF-κB in HEK-BLUE hTLR4 but not HEK-BLUE NULL cells. **(B)** SYQP showed no cytotoxicity. Cells were treated with SYQP for 24 h, and cell viability was evaluated by MTS assay. **(C)** SYQP pretreatment could not inhibit LPS-induced NF-κB activation in HEK-BLUE hTLR4 cells. Cell culture supernatants were mixed with QUANTI-BLUE substrate to test the level of SEAP, which represented the activity of NF-κB in the HEK-BLUE reporter cells. All of the data were normalized to the 1 μg/ml LPS-treated group, which represented the maximum response of the cells. All of the results are presented as the mean ± SD (*n* = 3).

HEK-BLUE hTLR4 cells were transgenic cells cotransfected with hTLR4, MD-2/CD14 coreceptor, and SEAP reporter genes. The SEAP reporter gene was controlled by the IL-12 P40 promoter with the NF-κB binding site. Stimulation of TLR4 on the cell membrane will activate NF-κB and result in the expression of SEAP. As shown in Figure 1A, when the concentration of SYQP was higher than 0.001 μg/ml, NF-κB was significantly activated in a dose-dependent manner, and when the concentration was higher than 10 μg/ml, the activation reached the plateau. The EC50 of SYQP stimulation in HEK-BLUE hTLR4 cells was 0.114 μg/ml. The results also show SYQP could not stimulate HEK-BLUE Null cells, which were only stably transfected with SEAP reporter gene but without hTLR4 and MD-2/CD14 coreceptor genes. The results of cell viability evaluation show SYQP has no cytotoxicity and proliferation promotion effect on both HEK-BLUE hTLR4 and HEK-BLUE Null cells (Figure 1B). Furthermore, SYQP pretreatment could not inhibit 1 μg/ml LPS-induced NF-κB activation in HEK-BLUE hTLR4 cells (Figure 1C).

### SYQP Stimulated Human TLR4 in PMA-Induced THP-1 Cells

**FIGURE 2:**
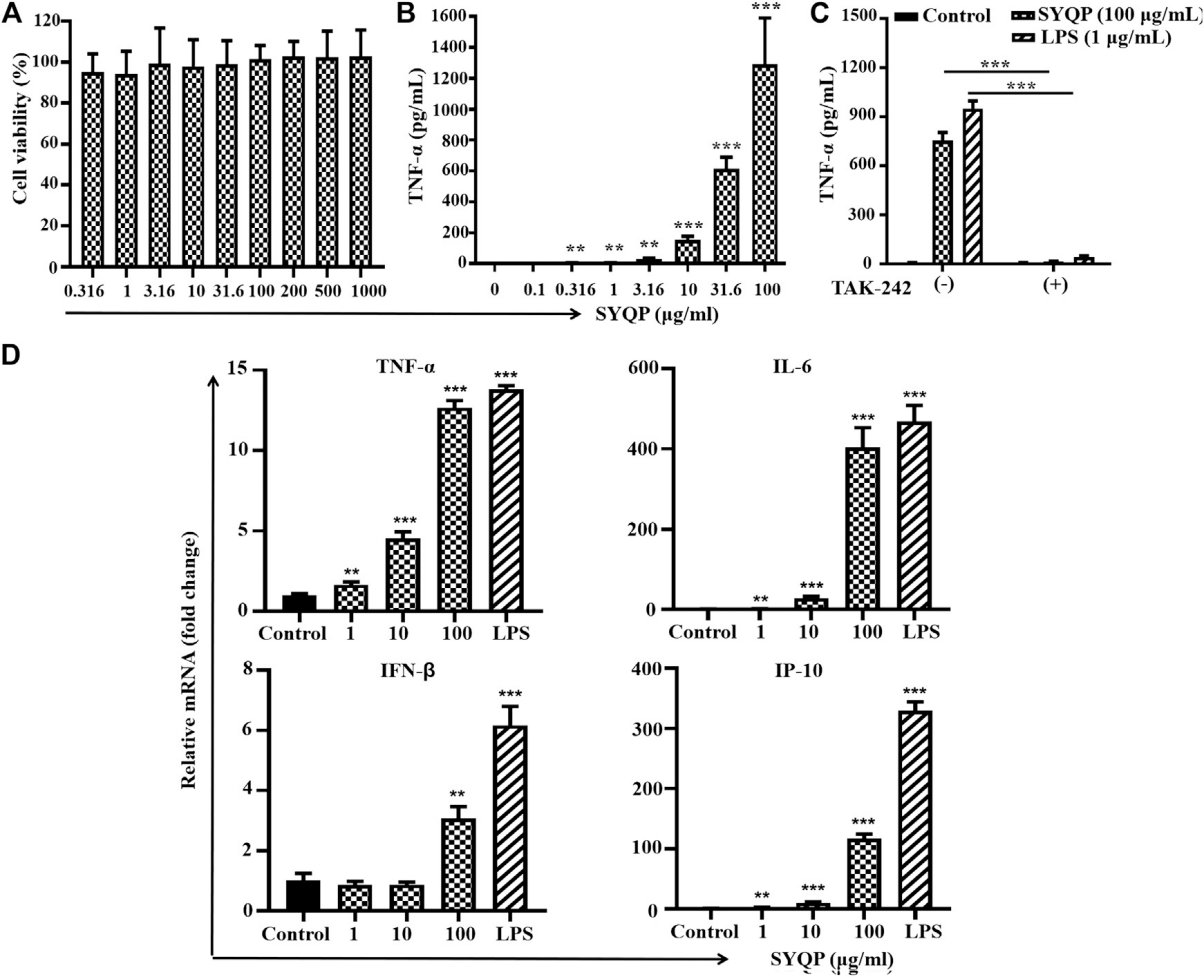
SYQP stimulated human TLR4 in PMA-induced THP-1 cells. **(A)** Effects of SYQP on viability of THP-1 cells. Cells were treated with SYQP for 24 h, and cell viability was evaluated by MTS assay. **(B)** SYQP increased the secretion of TNF-α from THP-1 cells. The cells were treated with SYQP at the indicated concentration for 24 h. Cell culture medium was collected, and the secretion levels of cytokines were detected by ELISA. ***p < 0.001 vs. control (the concentration of SYQP was zero). **(C)** TLR4 antagonist TAK-242 decreased the secretion of TNF-α promoted by SYQP. The cells were treated with 100 μg/ml SYQP alone or combined with TAK-242 for 24 h. LPS at 1 μg/ml was used as a positive control. Cell culture medium was collected, and the secretion levels of TNF-α were detected by ELISA. ***p < 0.001 vs. cells treated without TAK-242. All of the results are presented as the mean ± SD (*n* = 3). **(D)** SYQP induced mRNA expression of both MyD88-dependent and TRIF-dependent cytokines in THP-1 cells. The cells were treated with 1, 10, 100 μg/ml SYQP or 1 μg/ml LPS for 6 h. After that, mRNA was isolated, and the gene expression levels of cytokines were determined by real-time PCR. The results are presented as the mean ± SD (*n* = 3). **p < 0.01, ***p < 0.001 vs. control.

As HEK-BLUE hTLR4 is a transgenic cell line, to further confirm the human TLR4 stimulation effect of SYQP, we performed a similar experiment in human monocyte cell line THP-1 with a natural TLR4 signaling system. It was reported that 5 ng/ml PMA can stimulate THP-1 cells to differentiate into macrophage-like cells. CD14 expression on the cell membrane is increased, and the reaction of TLR4 signaling is good (Sun et al., 2009). Therefore, we used 5 ng/ml PMA-treated THP-1 cells for 48 h before drug treatment, and we found that THP-1 cells changed from suspended to adherent cells and showed the morphology of macrophages. To exclude the effect of SYQP on the growth of THP-1 cells, treatment with SYQP at 0.5–1,000 μg/ml for 24 h was performed, and cell viability was tested after treatment by using an MTT assay. As shown in Figure 2A, SYQP did not show any cytotoxicity in THP-1 cells even at a high concentration of 1,000 μg/ml. TNF-α is an important cytokine released by the activation of multiple signaling pathways, including TLR4. To determine whether SYQP can activate the TLR4 signaling pathway, we first observed the secretion of TNF-α from THP-1 cells. As shown in Figure 2B, SYQP could significantly increase TNF-α production in PMA-stimulated THP-1 cells. TAK-242 is an antagonist of both mouse and human TLR4. As TNF-α is released by the activation of multiple signaling pathways, we further used TAK-242 to determine whether the increased secretion of TNF-α induced by SYQP is due to specific TLR4 activation. As shown in Figure 2C, this increase can be almost completely abolished by TAK-242, which indicates the activation of TLR4 is at receptor level.

To determine whether SYQP activates both MyD88-dependent and -independent pathways, we detected the mRNA levels of MyD88 pathway-related (TNF-α, IL-6) and TRIF-related (IP-10, IFN-β) cytokines by real-time PCR after SYQP treatment. The mRNA levels of MyD88-dependent cytokines TNF-α and IL-6 and TRIF-dependent IP-10 and IFN-β induced by 1–100 μg/ml all increased significantly in the human cell line THP-1 (*p* < 0.01, Figure 2D).

### SYQP Stimulated Mouse TLR4 in RAW264.7 Cells

**FIGURE 3:**
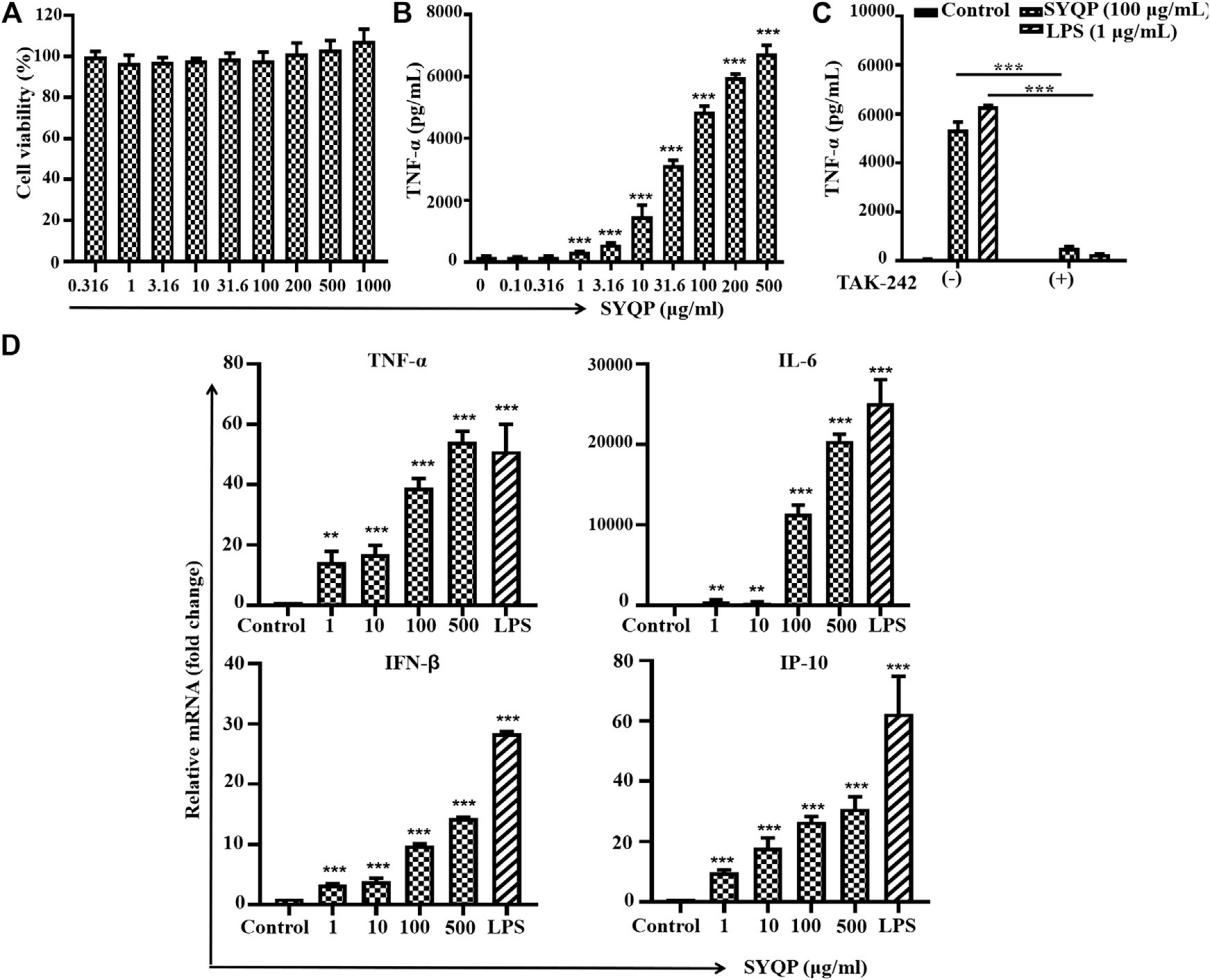
SYQP stimulated mouse TLR4 in RAW264.7 cells. **(A)** Effects of SYQP on the viability of RAW264.7 cells. **(B)** SYQP increased the secretion of TNF-α from RAW264.7 cells. The cells were treated with SYQP at the indicated concentration for 24 h. Cell culture medium was collected, and the secretion levels of cytokines were detected by ELISA. ***p < 0.001 vs. control. **(C)** TAK-242 decreased the secretion of TNF-α promoted by SYQP. ***p < 0.001 vs. cells treated with and cells treated without TAK-242. **(D)** SYQP upregulated mRNA expression of both MyD88- and TRIF-dependent cytokines in RAW264.7 cells. The cells were treated with 1, 10, 100, 500 μg/ml SYQP or 1 μg/ml LPS for 6 h. After that, mRNA was isolated, and the gene expression levels of cytokines were determined by real-time PCR. **p < 0.01, ***p < 0.001 vs. control. All of the results are presented as the mean ± SD (*n* = 3).

RAW264.7 cells were treated with SYQP for 24 h, and then the secretion of TNF-α in the supernatant was detected by ELISA reagent kit. As shown in Figure 3B, SYQP increased the secretion of TNF-α in a concentration-dependent manner. SYQP at 1 μg/ml began to increase the secretion significantly (*p* < 0.001). Treatment with SYQP 0.5–1000 μg/ml for 24 h was also performed to evaluate cell viability. As shown in Figure 3A, SYQP did not show any cytotoxicity and proliferation promotion effect on RAW264.7 cells. As shown in Figure 3C, a minimum amount of TNF-α (73.3 ± 6.4 pg/ml) was secreted when RAW264.7 cells were exposed to medium alone. However, when cells were incubated with SYQP (100 μg/ml) or LPS (1 μg/ml), the secretion of TNF-α was increased to 5,365 ± 314 or 6,311 ± 56 pg/ml, respectively. Notably, after cotreatment with 20 μg/ml TAK-242, the secretion of TNF-α significantly decreased again to 531 ± 68 or 277 ± 14 pg/ml, respectively (*p* < 0.001).

Further results show 1–500 μg/ml SYQP can increase the mRNA expression levels of all four cytokines in a concentration-dependent manner in RAW264.7 cells (*p* < 0.01, Figure 3D). Consistent with the effective concentration in Figure 3B, 1 μg/ml SYQP can significantly increase the mRNA expression of the MyD88-dependent cytokines TNF-α and IL-6 and TRIF-dependent cytokines IP-10 and IFN-β. SYQP at 500 μg/ml even showed higher activity than the positive control with 1 μg/ml LPS in terms of the mRNA expression of TNF-α.

### SYQP Activated TLR4 Without Selection of Downstream Pathways

**FIGURE 4:**
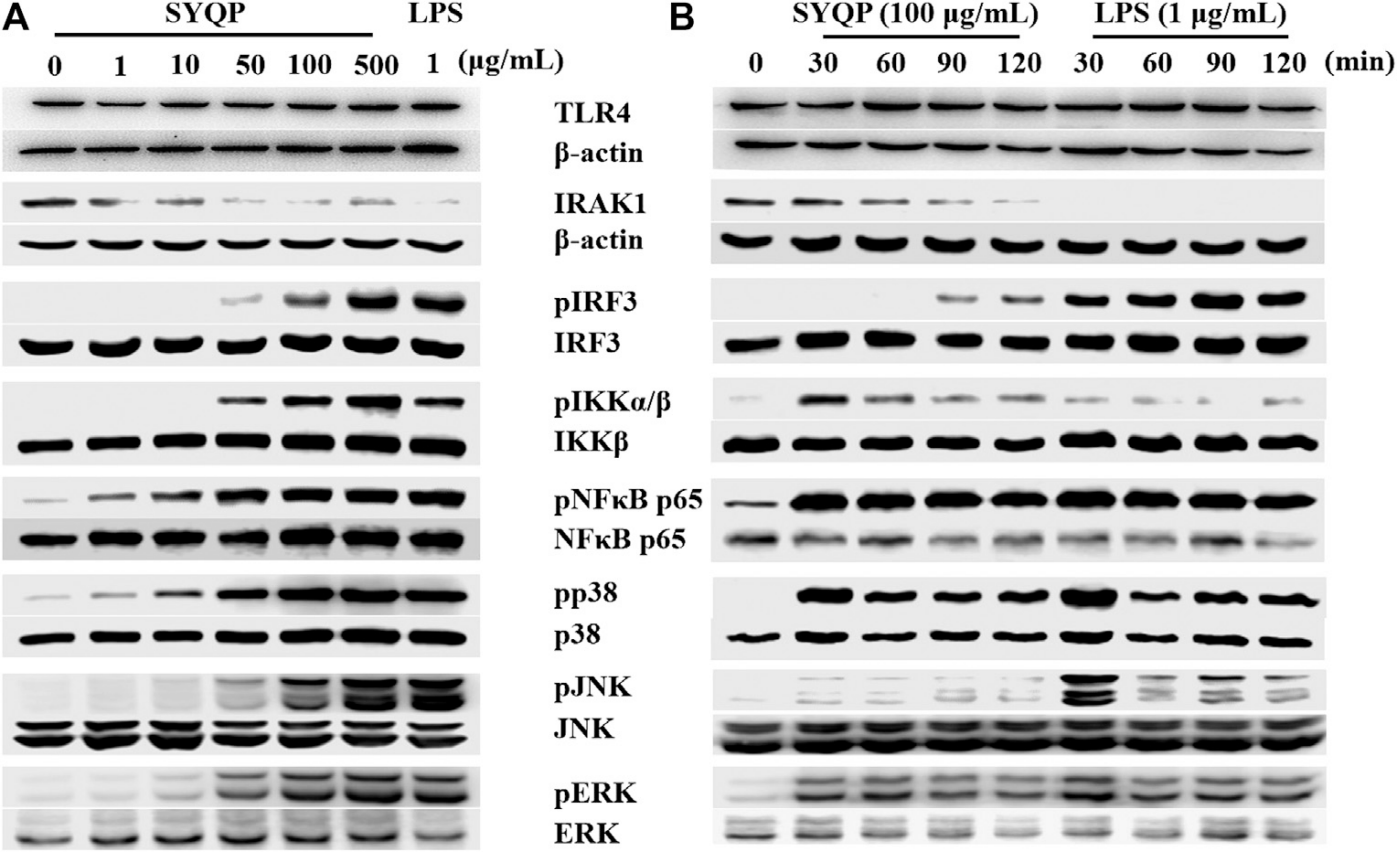
SYQP activated pathways downstream of TLR4 without selection. **(A)** RAW264.7 cells were treated with 1–500 μg/ml SYQP or 1 μg/ml LPS for 2 h (TLR4, IRAK1, IRF3) or 30 min (IKK, NF-κB, p38, JNK and ERK). **(B)** RAW264.7 cells were treated with 100 μg/ml SYQP or 1 μg/ml LPS for the indicated time points. The expression of molecules in the TLR4 pathways was determined by western blot analysis. The blot shown is a representative of one of three similar experiments. The densitometric analysis of the protein expression was shown in Supplementary Figure S2.

To further confirm whether SYQP could activate both the MyD88 and TRIF pathways, we detected the activation of two key molecules, IRAK1 and IRF3, which are respectively located in the MyD88 and TRIF pathways downstream of TLR4. As shown in Figure 4A, the SYQP concentration dependently decreased the expression of IRAK1 and increased the phosphorylation of IRF3. It is notable that the activation effect of SYQP 100 μg/ml was as strong as the positive control LPS (1 μg/ml). In addition, we observed the time-dependent effect of SYQP by treating with 100 μg/ml SYQP at the time points of 0, 30, 60, 90, and 120 min. We found that, at the time point of 90 min, IRAK1 decreased obviously and phosphorylation of IRF3 began to appear (Figure 4B). This activation time point is later than LPS, which shows obvious disappearance of IRAK1 and phosphorylation of IRF3 as early as 30 min. The densitometric analysis of the protein expression was shown in Supplemental Figure S2.

The expression of MyD88-dependent cytokines induced by TLR4 activation is dependent on further activation of the NF-κB or MAPK pathway. To understand if both pathways are activated by SYQP, we detected the protein expression of the related molecules after treatment with SYQP at 1–500 μg/ml. The results showed the SYQP concentration dose-dependently increased the phosphorylation of molecules in both pathways, such as IKK, NF-κB, p38, JNK, and ERK, and the activation time point was as early as 30 min (Figure 4).

### TLR4 Antagonist TAK-242 Abolished SYQP-Mediated TLR4 Signaling

**FIGURE 5:**
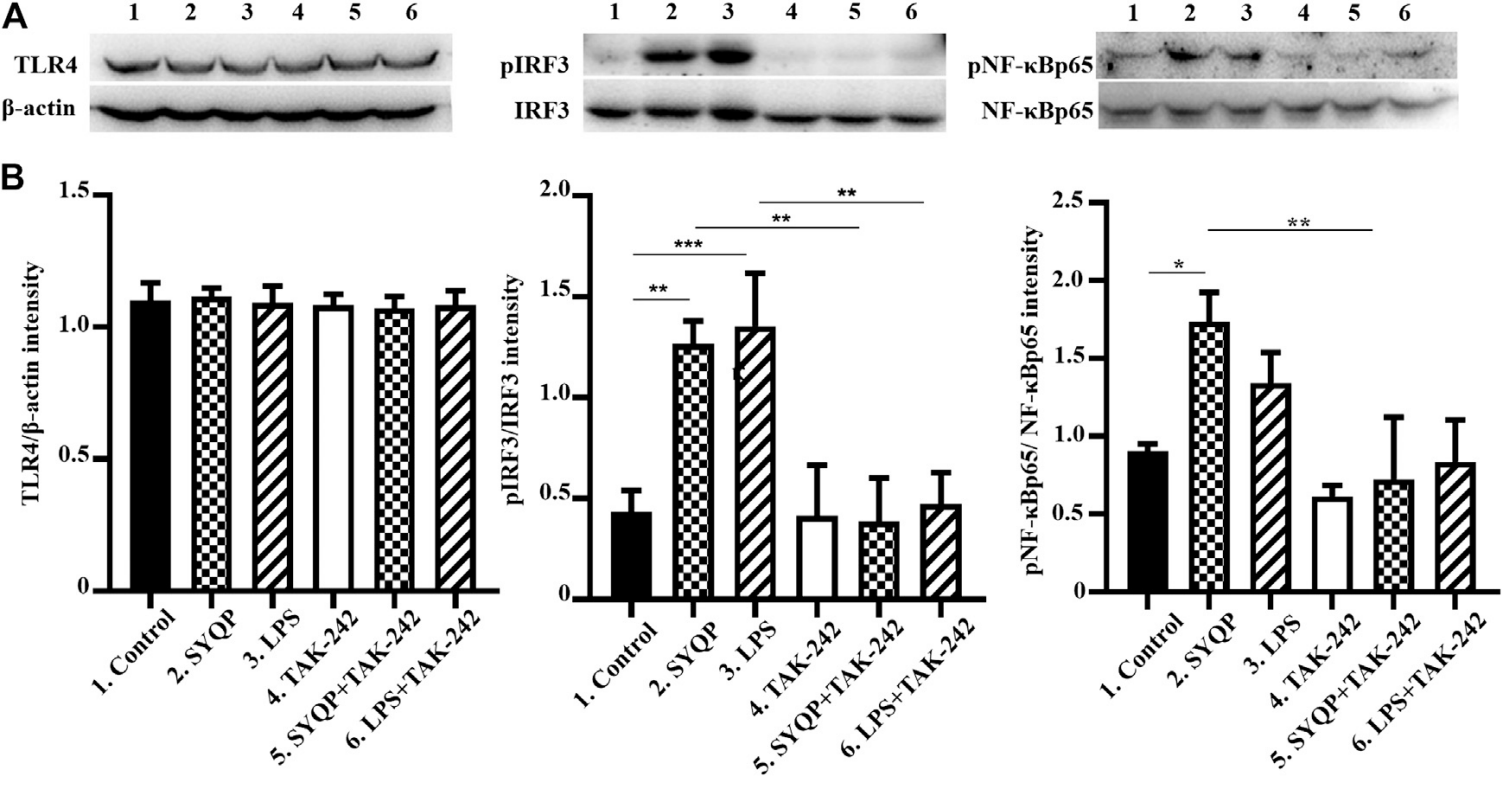
TAK-242 inhibited the phosphorylation of pIRF3 and pNF-κBp65 induced by SYQP. The RAW264.7 cells were pretreated with TAK-242 20 μg/ml for 30 min; then, SYQP 100 μg/ml or LPS 1 μg/ml were added into the cell supernatant alone or combined with TAK-242 for another 2 h. The cell lysates were analyzed by western blot. **(A)** The protein expression of TLR4, pIRF3, IRF3, NF-κBp65, and pNF-κBp65. **(B)** Densitometric analysis of the protein expression. The data are presented as the mean ± SD from three independent experiments.

To further confirm the TLR4-dependent macrophage activation of SYQP, we observed the effect of SYQP on RAW264.7 cells after pretreatment with TAK-242 for 30 min. The result showed that TAK-242 almost completely abolished SYQP induced phosphorylation of IRF3 and NF-κB (Figure 5). We also observed expression of TLR4. As shown in Figure 5, SYQP treated for 2 h or TAK-242 pretreatment for 30 min did not change the expression of TLR4.

### SYQP Enhances Immune Responses to Specific Antigen in C57BL/6 Mice

**FIGURE 6:**
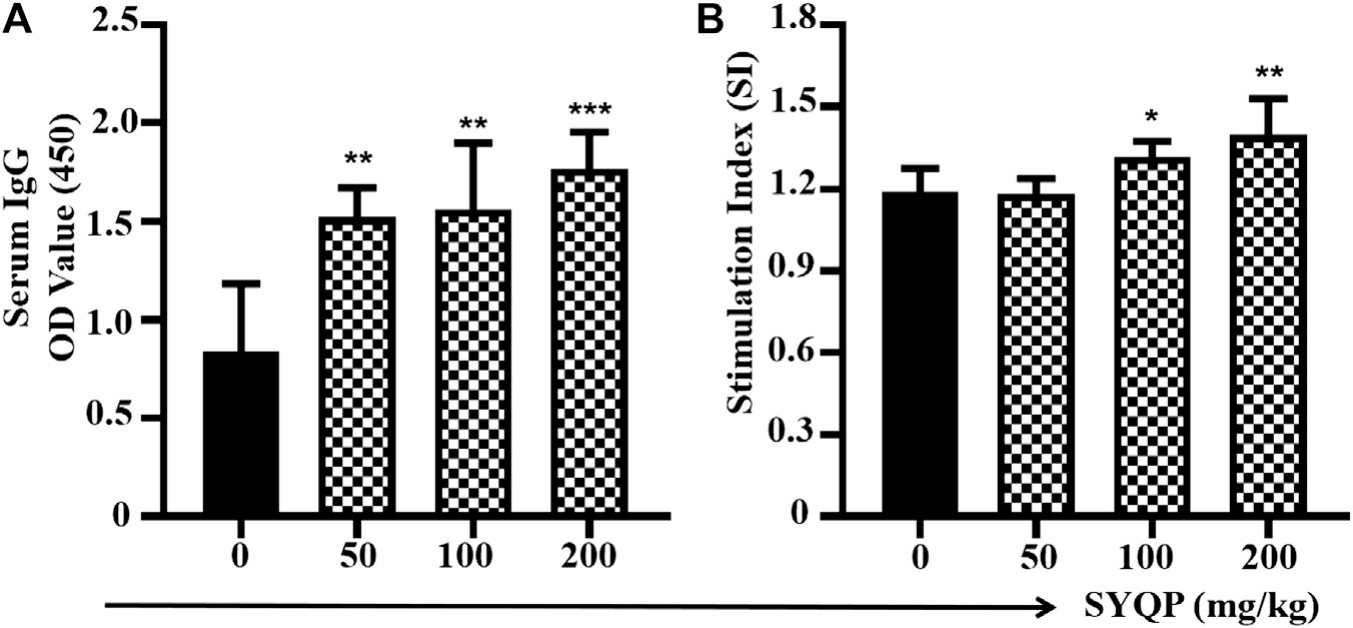
SYQP enhanced antigen-specific immune responses in OVA-immunized mice models. C57BL/6 mice were subcutaneously immunized with OVA (100 µg) at weeks 0 and 3. SYQP was given at 50, 100, and 200 mg/kg by intragastric administration once a day for four consecutive days before each immunization. Two weeks after the second immunization, blood samples were collected for measurement of OVA-specific IgG by ELISA. Splenocytes were harvested and exposed to OVA 100 μg/ml again for 48 h to determine antigen-specific lymphocyte proliferation by MTS assay. Stimulation index = (OD _OVA exposure_ − Blank)/(OD _OVA non-exposure_ − Blank). **(A)** SYQP enhanced specific serum IgG levels. **(B)** SYQP enhanced the spleen lymphocyte stimulation index. *p < 0.05, **p < 0.01, ***p < 0.001 vs. control (OVA-immunized mice without SYQP treatment).

Two weeks after the last immunization, the OVA-specific IgG antibody levels in the serum were tested by ELISA. As shown in Figure 6A, after coadministration with SYQP, the OVA-specific IgG antibody titers in C57BL/6 mice increased significantly compared with the mice immunized with OVA alone (*p* < 0.01). The spleen lymphocytes of immunized mice administered SYQP or saline were also separated and cultured *in vitro*. OVA at 100 μg/ml was added into the cell supernatant as a specific stimulator for 48 h. As shown in Figure 6B, SYQP at the doses of 100 and 200 mg/kg significantly promoted specific spleen cell proliferation (*p* < 0.05).

### SYQP Reduces Tumor Burden and Enhances the Spleen Weight of LLC-Bearing Mice

**FIGURE 7:**
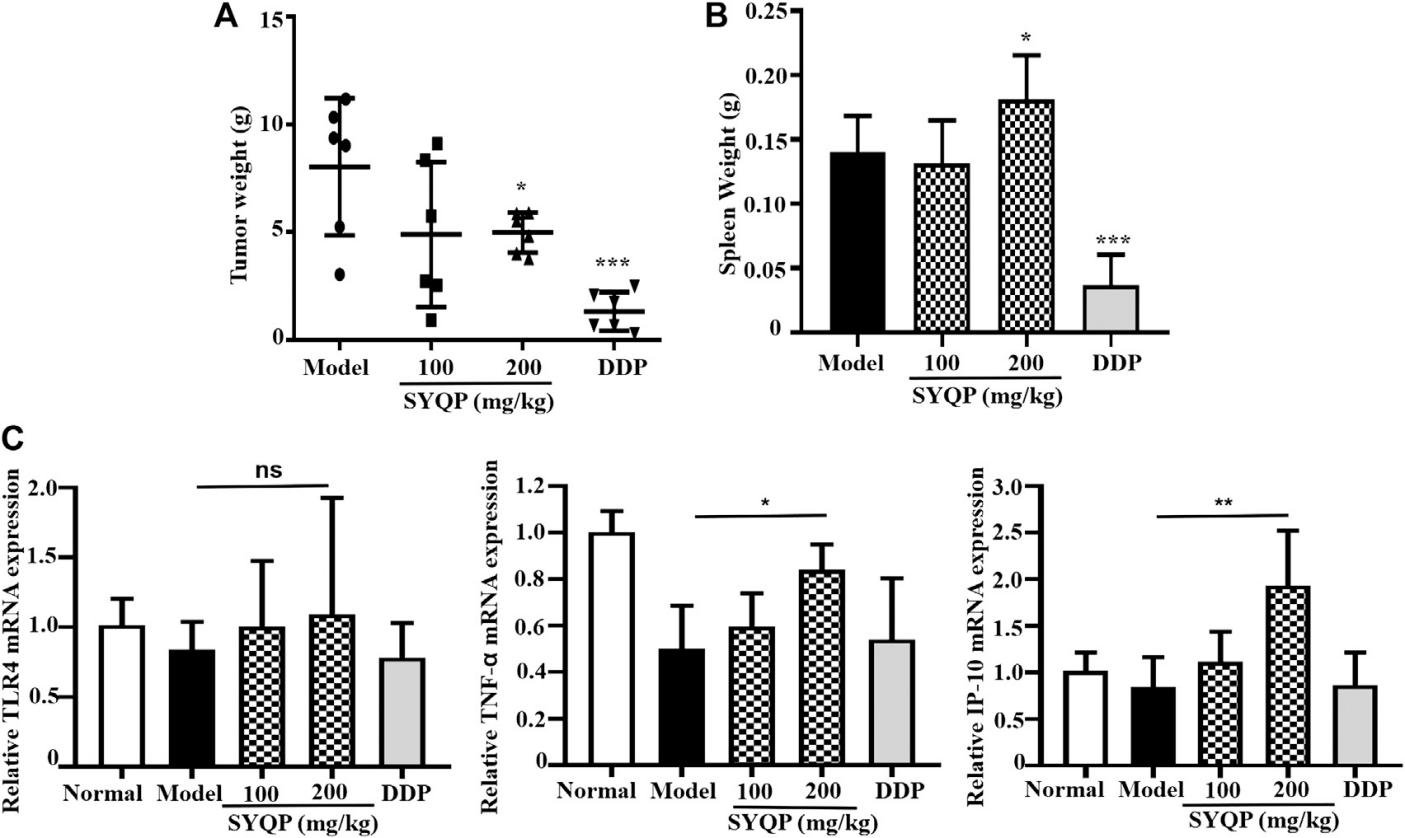
SYQP reduced tumor burden and enhanced spleen weight of LLC-bearing mice. C57BL/6 mice (*n* = 6) were injected s.c. with LLC tumor cell lines. Once palpable tumors were formed (day 7), mice were orally administered with SYQP (100, 200 mg/kg) or PBS (control) for seven consecutive days. DDP at 2 mg/kg was intraperitoneally injected once every other day and set as a positive control. On day 15, mice were killed, and tumor tissues and spleen were excised and weighed. **(A)** SYQP administration reduced the tumor burden of LLC-bearing mice. **(B)** SYQP administration increased the spleen weight of LLC-bearing mice. **(C)** The effect of SYQP on the mRNA expression of TLR4, TNF-α, and IP-10 in the spleen tissue. *p < 0.05, ***p < 0.001 vs. control.

To determine the *in vivo* antitumor effect of SYQP, C57/BL6 mice were challenged with the LLC cell line (s.c.). Mice with palpable tumors were orally administered SYQP or saline once a day for seven consecutive days. As shown in Figure 7A, SYQP treatment at 200 mg/kg significantly diminished the tumor weight (*p* < 0.05). Although 100 mg/kg SYQP also decreased the mean of the tumor weight, the SD value is large. Moreover, SYQP administration increased the spleen weight of LLC-bearing mice (Figure 7B).

### SYQP Increased the mRNA Expressions of TNF-α and IP-10 in the Spleen Tissue of LLC-Bearing Mice

To better understand the effect of SYQP on TLR4 pathway–related molecules *in vivo*, we detected the mRNA expression of TLR4, TNF-α, and IP-10 in the spleen tissue of mice. The results showed that there was no significant difference in TLR4 expression among normal control, LLC-bearing control, DDP, and SYQP administrated mice. However, compared with LLC-bearing model control mice, administration of SYQP 200 mg/kg could increase the mRNA expression of TNF-α and IP-10 (*p* < 0.05, Figure 7C).

### Exclusion of Endotoxin Contamination

LAL kit was used to test the endotoxin concentration in the SYQP samples. The concentration of endotoxin was lower than 0.05 EU/ml, even in 1 mg/ml SYQP (data not shown), which suggests no contamination of LPS in the SYQP samples used in this study. The natural peptide, Polymyxin B (PMB), is a potent antibiotic that binds to and neutralizes LPS (Su et al., 2012). Pretreatment of PMB with samples will help to determine whether endotoxin contamination exists. To exclude the endotoxin contamination of SYQP in this study, we further used 5 μg/ml PMB for detection in HEK-BLUE hTLR4 cells. As shown in Supplemental Figure S3, the TLR4 activation of LPS (1 μg/ml) was significantly inhibited by the pretreatment of PMB (*p* < 0.001). However, the TLR4 activation of SYQP solution (100 μg/ml) was not affected by PMB, which further confirmed no contamination of LPS in the SYQP samples.

## Discussion

The main findings of the present study are that SYQP can stimulate both human and mouse TLR4 in macrophage cell lines and show immune adjuvant and anticancer activity *in vivo*. HEK-BLUE hTLR4 is a good tool for studying TLR4 signaling and is cotransfected with hTLR4, MD-2/CD14 coreceptor, and SEAP reporter genes. Stimulation of TLR4 on the cell membrane will result in the activation of the downstream transcript of NF-κB and induce the secretion of SEAP. Therefore, the level of SEAP in the cell supernatant can represent the degree of activation of NF-κB as well as the stimulation of TLR4. HEK-BLUE Null is a cell line only stably transfected with the SEAP reporter gene but without hTLR4 and MD-2/CD14 coreceptor genes. Our results show SYQP treatment increased NF-κB in HEK-BLUE hTLR4 but not HEK-BLUE Null cells in a concentration-dependent manner, which suggests the TLR4 stimulation of SYQP. Some receptor agonists may also show antagonist activity upon cotreatment with another strong agonist. Therefore, we observed NF-κB activation after using different concentrations of SYQP cotreatment with 1 μg/ml LPS. The result shows SYQP could not inhibit LPS-induced TLR4 stimulation in HEK-BLUE hTLR4 cells (Figure 1C). In a previous study, we found SYQP can decrease the serum inflammatory cytokine levels in LPS-induced inflammatory mice model (Zhu et al., 2020). According to the results of this study, the anti-inflammation effect of SYQP does not occur through competition with LPS for TLR4 binding sites. It may occur through some other negative feedback mechanism as reported previously (Afonina et al., 2017).

As HEK-BLUE hTLR4 is a transgenic cell line with an artificial TLR4 system, we next used PMA-stimulated human monocyte THP-1 cells with natural TLR4 signaling pathways to further confirm the TLR4 stimulation effect of SYQP. TNF-α is an important inflammatory cytokine secreted after NF-κB activation in various types of cells, especially monocytes. It was first identified as a factor with an anticancer effect (Idriss and Naismith, 2000). Although it was proven that chronic inflammation induced by TNF-α can mediate most chronic diseases, acute inflammation has definite anticancer potential (Boushehri Shetab and Lamprecht, 2018). Our results show SYQP can stimulate the secretion of TNF-α in PMA-treated THP-1 cells. The activation occurs immediately, and the maximum effect is close to that of 1 μg/ml LPS, which indicates the ability of SYQP to induce acute inflammation. The secretion of TNF-α can be stimulated by various signals such as ligands of TLRs and even TNF-α itself through their corresponding receptors (Idriss and Naismith, 2000). We next used the TLR4 antagonist TAK-242 to determine whether SYQP could also increase the secretion of TNF-α by activating TLR4 at receptor level. TAK-242 is a small molecule inhibiting the production of LPS-induced inflammatory mediators by binding to the intracellular domain of TLR4 (Matsunaga et al., 2011). In this study, we found the increased secretion of TNF-α promoted by SYQP was almost completely abolished by TAK-242, which further confirmed the human TLR4 stimulation effect of SYQP (Figure 2C). Polymyxin B (PMB) is a potent antibiotic that binds to and neutralizes LPS. It is a decapeptide cyclic cationic antibiotic containing lipophilic and hydrophilic groupment (lipophobic) that binds to lipid A, the major component of the endotoxin (Cardoso et al., 2007). In this study, the results showed that TAK-242 could block SYQP-induced activation of THP-1 or HEK-BLUE hTLR4 cells, but PMB could not (Figure 2C; Supplemental Figure S2). This further confirmed that SYQP acted through TLR4 rather than lipid A structure. It was reported that some agonists show different stimulating activities between mouse and human TLR4 (Marshall et al., 2016; Nativel et al., 2017). Accordingly, we also used the mouse macrophage cell line RAW264.7 to observe the effect of SYQP on mouse TLR4. The results are similar to those in THP-1 cells, which indicates SYQP is also a strong mouse TLR4 agonist (Figure 3C).

It is known that stimulation of TLR4 will subsequently trigger both the MyD88- and TRIF-dependent pathways. Activation of the MyD88 pathway induces the mRNA expression of a variety of MyD88-dependent inflammatory cytokines such as TNF-α and IL-6. Activation of the TRIF pathway induces the mRNA expression of TRIF-dependent cytokines such as IP-10 and type-Ⅰ interferon (Medzhitov, 2001). Studies show that some TLR4 agonists have TRIF-bias characteristics (Mata-Haro et al., 2007). To further understand the TLR4 activation characteristics of SYQP, we examined the gene expression of cytokines and chemokines of both the MyD88- and TRIF-dependent pathways. Our results showed that SYQP could increase the mRNA expression of TNF-α, IL-6, IP-10, and IFN-β, which suggests that it can activate both the MyD88 and TRIF pathways without pathway bias (Figures 2D, 3D). IRAK1 and IRF3 are two key molecules that mediate the MyD88 and TRIF pathways, respectively. The disappearance of IRAK1 results from polyubiquitination is an important sign of activation of the MyD88 pathway (Smith et al., 2009). Phosphorylation of IRF3 is an important sign of activation of the TRIF pathway (Mata-Haro et al., 2007). Our results show SYQP decreased the expression of IRAK1 and increased the phosphorylation of IRF3 in a concentration- and time-dependent manner. These results further confirmed that SYQP activates both the MyD88 and TRIF pathways without pathway bias (Figure 4).

The expression of proinflammatory genes induced by the activation of TLR4 depends on the intranuclear translocation of NF-κB and AP-1 transcripts (Kawai and Akira, 2006). The phosphorylation of IКB kinase (IKK) can induce the phosphorylation and degradation of NF-κB inhibitor (IКB), which subsequently allows NF-κB to be released to the nucleus. The phosphorylation of MAPK, including c-Jun N-terminal kinase (JNK), p38, and extracellular signal-regulated kinase 1/2 (ERK1/2), can activate AP-1. Caglar Cekic et al. reported selective activation of the p38 MAPK pathway by a synthetic TLR4 agonist (Cekic et al., 2009). To further explore whether SYQP has a selective effect on the NF-κB and MAPK pathways, we detected the phosphorylation of IKK, NF-κB p65, JNK, p38, and ERK. The results showed that SYQP increased the phosphorylation of IKK, NF-κB p65, JNK, p38, and ERK in a concentration-dependent way. In addition, the phosphorylation increased upon SYQP treated for 30 min. These effects are similar to those of LPS and some other TLR4 agonists reported previously (Cekic et al., 2009).

The activation of the TLRs may break tumor-induced immune tolerance and result in antitumor effects (Boushehri Shetab and Lamprecht, 2018). A TLR agonist can be used alone or as a vaccine adjuvant to potentiate the immunogenicity of tumor antigens and enhance the innate and adaptive immune responses against cancer. Although chronic inflammation in the tumor microenvironment increases the levels of growth and survival factors and promotes the progression of tumors, the induction of acute inflammation induced by a strong TLR agonist and cancer vaccine has proven to be a potential strategy for the treatment of cancer (Adams, 2009; Wang et al., 2011; Adams et al., 2012). TLR4 is the only TLR with the ability to induce both MyD88- and TRIF-dependent pathways, which will maximize the immunostimulatory potential of immune cells (Xiao et al., 2012). Activation of TLR4 can increase the expression of costimulator molecules on APC cells and improve antigen presentation, resulting in the activation of cytotoxic T lymphocytes and killing of cancer cells. Therefore, TLR4-based cancer immunotherapy has received great attention. As SYQP shows the characteristics of a strong TLR4 agonist, we then further observed its immune adjuvant and antitumor effects in a mouse model. Our results show SYQP can enhance OVA-induced antigen specific spleen lymphocyte proliferation and serum antibody production in mice. Macrophage is an important kind of APC which can activate B cells differentiating into plasma cells through T-cell-dependent (TD) or T-cell-independent (TI) manner. In TD manner, macrophages can take up antigens and present them to T helper cells. Activated T helper cells will then further interact with B cells and result in plasma cells differentiation. In TI manner, macrophage-derived factors such as IP-10 directly induce B cells differentiating into the plasma cells (Xu and Banchereau, 2014). The activation of T cells and the differentiation of B cells will result in the proliferation of lymphocyte proliferation and antibody production. In this study, the effect of SYQP on spleen lymphocyte proliferation and serum antibody production in OVA-immunized mouse model was consistent with the activation of TLR4 signaling *in vitro*. The stimulation of TLR4 on APCs like macrophages, DCs and B cells may increase the uptake and present of OVA, promote the secretion of cytokines such as IP-10, then further stimulate T cells activation and B cells differentiation. Although each kind of cancer has a large number of potential tumor antigens, these cancer antigens are not strong enough to induce the specific T- and B-cell activation (Banchereau and Palucka, 2005). Therefore, immune adjuvant is beneficial in cancer immunotherapy. The results in this study show SYQP can enhance antigen specific immune responses in OVA-immunized mouse model, which indicates SYQP is potentially useful in cancer immunotherapy.

In order to observe the antitumor effect of SYQP, we chose LLC mouse model for experiment and observation. One reason why we chose this mouse model is that the plant of *Tetrastigma hemsleyanum* Diels et Gilg is commonly used in the treatment of respiratory diseases in clinic. The other reason is that we speculate that SYQP plays an antitumor role by activating TLR4 and activating the immune system. Therefore, mice with complete immune system instead of human tumor and nude mice should be used in this study. Lewis lung cancer is one of the most commonly used tumor types which can be transplanted in normal mice. The results in this study show SYQP can obviously reduce tumor burden at the concentration of 200 mg/kg. DDP is a chemotherapeutic drug which is known for good effect in solid tumor such as testicular, ovarian, and lung cancer. So we used DDP as a positive control to see the tumor growth inhibition of SYQP. Interestingly, although we found both SYQP and positive control DDP show tumor inhibition, their effects on spleen are very different. DDP decreased the spleen weight dramatically while SYQP did not decrease the spleen weight and even increased it at a higher concentration (200 mg/kg). DDP can bind with DNA to create DNA lesions; block the production of DNA, mRNA, and proteins; arrest DNA replication; and activate several transduction pathways which finally led to necrosis or apoptosis (Ghosh, 2019). Because of its direct cytotoxicity, DDP show obvious nephron-, hepato-, and gastrointestinal toxicity in clinical application. In this study, the obvious decrease of spleen weight may be due to the lymphocyte cytotoxicity of DDP which result in the induction of spleen cell necrosis and apoptosis. It is known that LPS can act as a mitogen and stimulate the B-cell proliferation through TLR4 activation. The effect of SYQP on spleen weight may be due to its similar effect on LPS, which stimulates TLR4 receptor of B cells and promotes cell proliferation. The effect of SYQP on spleen weight indicates that the combination of the two drugs may be beneficial to minimize the side effect of DDP. The activation of TLR4 on B cells may result in the expression of TLR4-related cytokines. Therefore, we also tested the mRNA expression of TLR4-related cytokines in the spleen tissue. The result shows SYQP increased the secretion of IP-10 and TNF-α, which is consistent with the results in macrophage cell lines. IP-10 and TNF-α are two important cytokines in antitumor immunity. IP-10 is a key chemokine recruiting and activating effector T cells. TNF-α can induce DC maturation and migration, which in turn result in the proliferation of Th1 cells. TNF-α is also a crucial effector molecule in CD8^+^ T cells and natural killer cells (Boushehri Shetab and Lamprecht, 2018). As tumor-bearing organisms are usually in a state of immunosuppression, the promotion of the secretion of IP-10 and TNF-α may activate the immune response and exhibit antitumor effect (Kaufman and Disis, 2004).

In conclusion, the present study shows that SYQP can activate the TLR4 signaling pathway at the receptor level and shows TLR4 agonist characteristics in both mouse and human macrophage cell lines. SYQP also enhances immune responses and shows immune adjuvant and anticancer activities in mice. This study provides a basis for the development and utilization of SYQP as a new TLR4 agonist in the future.

## Data Availability

The raw data supporting the conclusions of this article will be made available by the authors, without undue reservation.
