# Supplementary material for: Polysaccharide Isolated From Tetrastigma hemsleyanum Activates TLR4 in Macrophage Cell Lines and Enhances Immune Responses in OVA-Immunized and LLC-Bearing Mouse Models
Source: Front Pharmacol. 2021 Mar 24;12:609059. doi: 10.3389/fphar.2021.609059 (PMC8024652; doi:10.3389/fphar.2021.609059)
Supplement: Supplementary file 1 [file table1.doc]

| **Table S1 The sequences of primers used in Real-time PCR analysis** | | | |
| --- | --- | --- | --- |
| Species | Gene | Primer | Sequence (5' - 3') |
| Human | GAPDH | FW | ACAACTTTGGTATCGTGGAAGG |
|  |  | RV | GCCATCACGCCACAGTTTC |
|  | TNF-α | FW | CCTCTCTCTAATCAGCCCTCTG |
|  |  | RV | GAGGACCTGGGAGTAGATGAG |
|  | IL-6 | FW | ACTCACCTCTTCAGAACGAATTG |
|  |  | RV | CCATCTTTGGAAGGTTCAGGTTG |
|  | IFN-β | FW | GCTTGGATTCCTACAAAGAAGCA |
|  |  | RV | ATAGATGGTCAATGCGGCGTC |
|  | IP-10 | FW | GTGGCATTCAAGGAGTACCTC |
|  |  | RV | TGATGGCCTTCGATTCTGGATT |
| Mouse | GAPDH | FW | CATCACTGCCACCCAGAAGACT |
|  |  | RV | GACACATTGGGGGTAGGAACAC |
|  | TNF-α | FW | CGAGTGACAAGCCTGTAGCCC |
|  |  | RV | GGGCAGCCTTGTCCCTTGA |
|  | IL-6 | FW | AGTTGCCTTCTTGGGACTGA |
|  |  | RV | TTCTGCAAGTGCATCATCGT |
|  | IFN-β | FW | CAGCTCCAAGAAAGGACGAAC |
|  |  | RV | GGCAGTGTAACTCTTCTGCAT |
|  | IP-10 | FW | CCAAGTGCTGCCGTCATTTTC |
|  |  | RV | GGCTCGCAGGGATGATTTCAA |
