# Supplementary figures and images for: Polysaccharide Isolated From Tetrastigma hemsleyanum Activates TLR4 in Macrophage Cell Lines and Enhances Immune Responses in OVA-Immunized and LLC-Bearing Mouse Models
Source: Front Pharmacol. 2021 Mar 24;12:609059. doi: 10.3389/fphar.2021.609059 (PMC8024652; doi:10.3389/fphar.2021.609059)

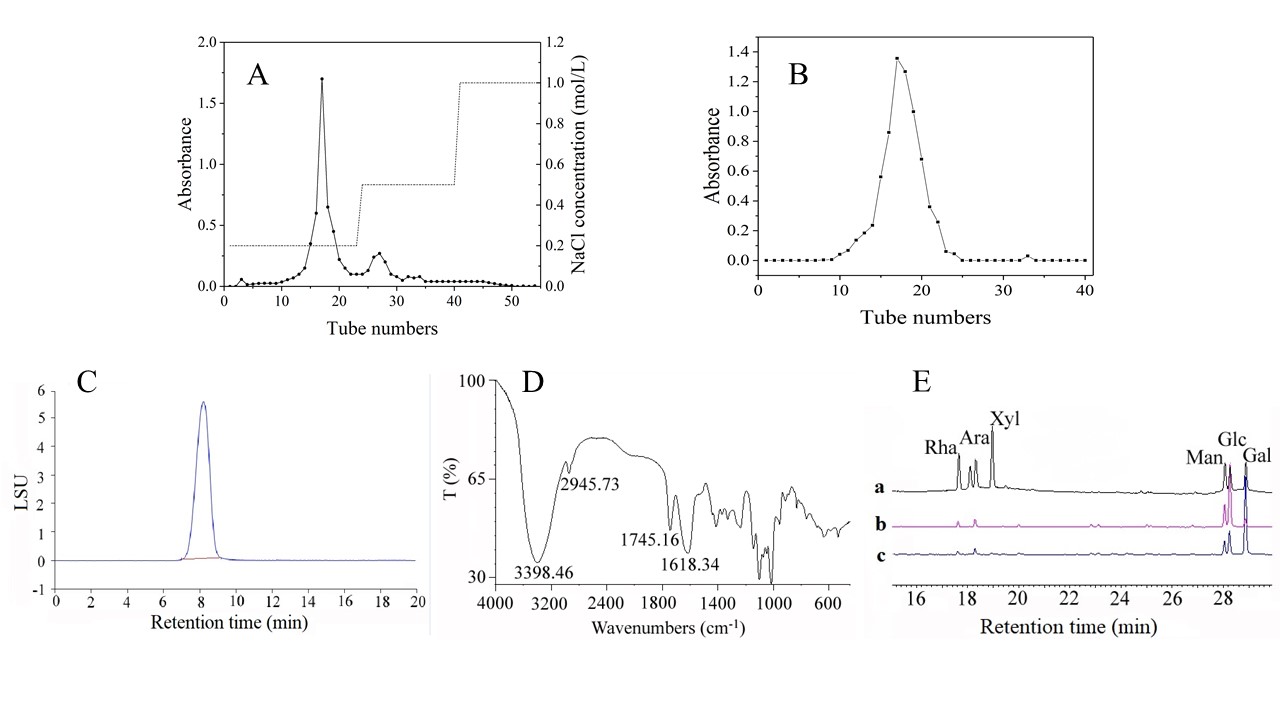

Supplement: Supplementary file 2 [file image1.jpeg]

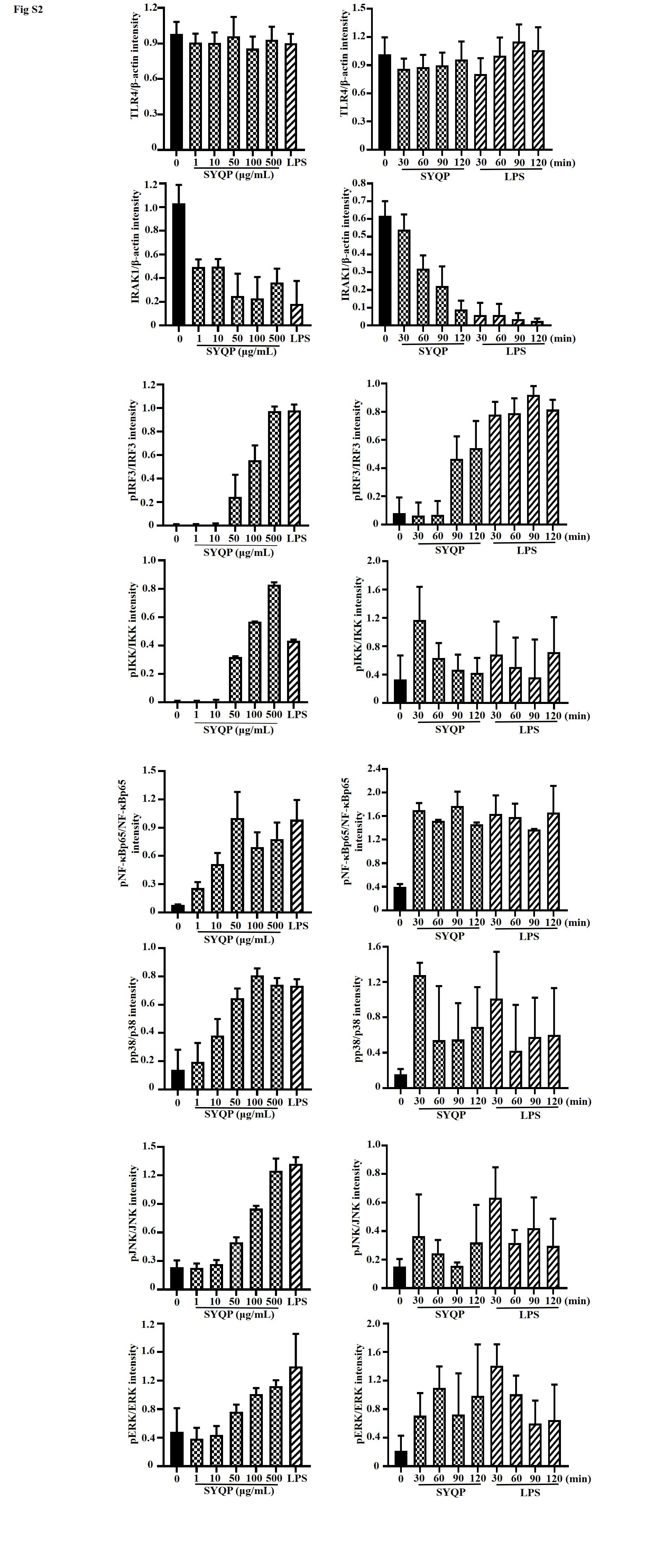

Supplement: Supplementary file 3 [file image2.jpeg]
